# Age-related defects in autophagy alter the secretion of paracrine factors from bone marrow mononuclear cells

**DOI:** 10.18632/aging.203127

**Published:** 2021-06-04

**Authors:** Azadeh Yeganeh, Faisal J. Alibhai, Stephanie W. Tobin, Fievel Lim, Jun Wu, Shuhong Li, Richard D. Weisel, Ren-Ke Li

**Affiliations:** 1 Toronto General Hospital Research Institute, University Health Network, Toronto, Canada; 2 Department of Surgery, Division of Cardiac Surgery, University of Toronto, Toronto, Canada

**Keywords:** autophagy, aging, bone marrow, fibroblasts, secretome

## Abstract

Bone marrow mononuclear cell therapy improves cardiac repair after myocardial infarction (MI), in-part through signaling to resident cardiac cells, such as fibroblasts, which regulate scar formation. The efficacy of cell therapy declines with age, as aging of both donor and recipient cells decreases repair responses. Autophagy regulates the microenvironment by both extracellular vesicle (EV)-dependent and independent secretion pathways. We hypothesized that age-related autophagy changes in bone marrow cells (BMCs) alter paracrine signaling, contributing to lower cell therapy efficacy. Here, we demonstrate that young Sca-1^+^ BMCs exhibited a higher LC3II/LC3I ratio compared to old Sca-1^+^ BMCs, which was accentuated when BMCs were cultured under hypoxia. To examine the effect on paracrine signaling, old cardiac fibroblasts were cultured with conditioned medium (CM) from young and old Sca-1^+^ BMCs. Young, but not old CM, enhanced fibroblast proliferation, migration, and differentiation, plus reduced senescence. These beneficial effects were lost when autophagy or EV secretion in BMCs was blocked pharmacologically, or by siRNA knockdown of *Atg7*. Therefore, both EV-dependent and -independent paracrine signaling from young BMCs is responsible for paracrine stimulation of old cardiac fibroblasts. *In vivo*, bone marrow chimerism of old mice with young BMCs increased the number of LC3b^+^ cells in the heart compared to old mice reconstituted with old BMCs. These data suggest that the deterioration of autophagy with aging negatively impacts the paracrine effects of BMCs, and provide mechanistic insight into the age-related decline in cell therapy efficacy that could be targeted to improve the function of old donor cells.

## INTRODUCTION

The number of aged individuals is increasing globally, resulting in an urgent need to address the escalating incidence of cardiovascular disease, particularly heart failure. Improving heart function in response to ischemic injury can slow the progression to heart failure and improve quality of life for aged individuals. Among the various approaches which improve cardiac repair following ischemic injury (myocardial infarction, MI), bone marrow mononuclear cell therapy has shown promising pre-clinical results [1, 2]. Bone marrow cells (BMCs) improve vascularization in ischemic heart disease [3] but do not restore cardiac muscle cells lost after MI [4]. Importantly, their benefits are generally mediated by the short-term secretion of paracrine factors within the infarcted myocardium. Unfortunately, the success of cell therapy observed in animal models has not been matched in clinical trials. One of the possible causes for the limited success of autologous cell transplantation is that patients are often older, therefore these donor cells cannot stimulate repair processes effectively. In comparison, most preclinical studies use young donor cells, which do not accurately represent the scenario in clinical trials [2]. Previous studies have demonstrated that instead of injecting BMCs into the infarcted myocardium, activating their recruitment may improve the benefits being achieved [5]. Li and colleagues demonstrated that repopulating the bone marrow (BM) of old mice with young BM stem cells (by BM transplant) resulted in the homing of BMCs to the myocardium. These cardiac resident, BM-derived cells were associated with improved cardiac function of the aged heart following an MI [6], suggesting an age-dependent role of BMCs in cardiac repair.

Autophagy is an evolutionarily conserved process which preserves cellular homeostasis and survival via protein recycling [7–9], but is also involved in paracrine signaling [10, 11]. An age-related autophagy decline in different organs, such as the brain, heart, muscle, and kidney, has been reported [12]. Inhibition of autophagy promotes aging [13, 14] while activation of autophagy diminishes the aging phenotype [15]. We hypothesized that age-related defects in autophagy would affect the secretome of BMCs, and thus impact paracrine signaling to resident cardiac cells. In the current study, we investigated the role of aging on autophagy and paracrine signaling between young or old BMCs and cardiac fibroblasts from old mice (20 months old). Cardiac fibroblasts were chosen as they serve a fundamental role in tissue repair and scar formation after MI. With aging, however, cardiac fibroblasts are less responsive to TGF-β mediated myofibroblast conversion, compromising collagen deposition and overall scar integrity, and therefore leading to heart failure [16]. To prevent infarct thinning and dilatation after myocardial injury, we postulated that autophagy may alter BMC signaling to influence cardiac fibroblasts in an age-dependent manner. In this current study, we demonstrate that young BMCs secrete factors which enhance the functions of old cardiac fibroblasts, and these paracrine effects were abolished when autophagy in BMCs was blocked via pharmacological inhibitors or siRNA. Additionally, loss of extracellular vesicle (EV) secretion also reduced overall cardiac fibroblast functional rejuvenation. These benefits were correlated with TGF-β1 levels in BMCs, identifying a mechanism whereby defects in donor cell autophagy alter paracrine stimulation of cardiac fibroblasts.

## RESULTS

### Conditioned medium from young Sca-1^+^ cells improves old cardiac fibroblast function

**Figure 1:**
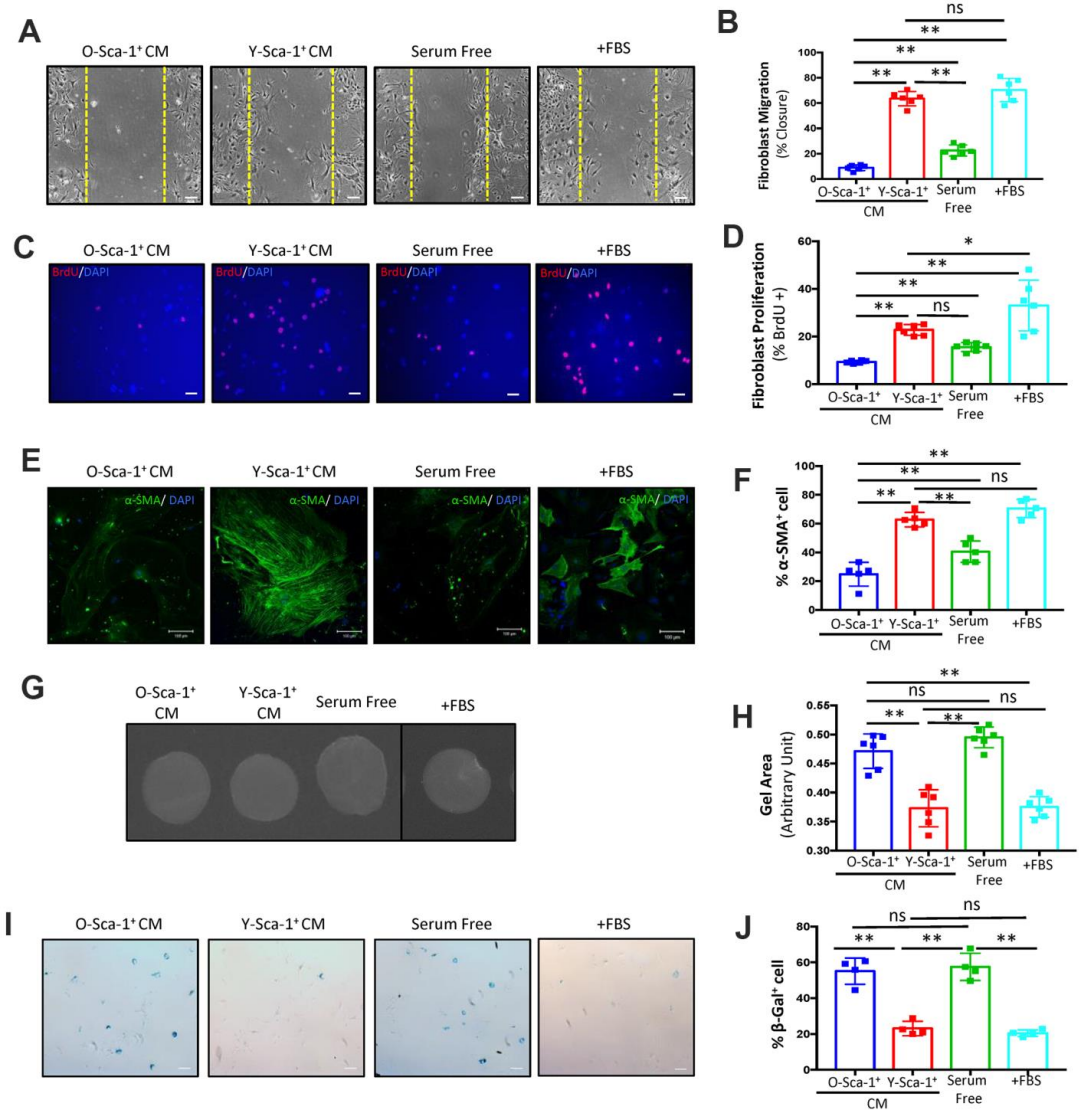
**Conditioned media from Y-Sca-1^+^ BMCs improves functional and age-related deficits in old cardiac fibroblasts.** (**A**) Representative images from scratch wound assay of old fibroblasts, treated with conditioned media (CM), from Y-Sca-1^+^ and O-Sca-1^+^ bone marrow cells (BMCs) for 48 hours. Dashed yellow line indicates the wound edge at 0 hours. After 48 hours, the closing distances were measured (**B**) (n=6). (**C**) Representative images from proliferation assay, after old fibroblasts were treated with CM from Y-Sca-1^+^ and O-Sca-1^+^ BMCs for 24 hours. BrdU is stained in red, and nuclei stained in blue. (**D**) Percentage of BrdU^+^ cells, normalized to total cell number. (**E**) Representative images of senescence assay (β-galactosidase^+^), after old fibroblasts were treated with CM from Y-Sca-1^+^ and O-Sca-1^+^ BMCs for 48 hours. (**F**) Percentage of SA-β-gal^+^ cells, normalized to total cell number (n=4). (**G**) Representative images of gels from gel contraction assay, after old fibroblasts were treated with CM from Y-Sca-1^+^ and O-Sca-1^+^ BMCs for 48 hours. (**H**) Gel area was measured using ImageJ (n=6). (**I**) Immunofluorescent staining for α-SMA was performed on old cardiac fibroblasts, after treatment with CM from Y-Sca-1^+^ and O-Sca-1^+^ BMCs for 48 hours. α-SMA is stained in green, and nuclei in blue. (**J**) Percentage of α-SMA^+^ cells, relative to total cell number (n=5). Scale bars represent 100 μm, unless otherwise stated. Data analysis was by one-way ANOVA. n=5-6; *p≤0.05, **p≤0.01; ns: not statistically significant.

To investigate the effect aging has on BMC paracrine signaling, stem and progenitor cells were isolated from BM of young (Y) and old (O) mice based on the cell surface marker, Sca-1^+^. Cells were then cultured under normoxic or hypoxic conditions for 24 hours, as previous studies have shown this activates several cell pathways, including paracrine signaling [17]. Hypoxia induced the expression of *Vegf* and *Tgf-β1* in young but not old Sca-1^+^ BMCs, indicating putative age-related differences in the secretome of BMCs at the transcriptional level (p<0.01) (Supplementary Figure 1A, 1B). Hypoxic conditions were used to generate all conditioned media in subsequent experiments. To investigate the role of secreted factors from young and old Sca-1^+^ BMCs on cardiac fibroblast function, conditioned medium (CM) was collected from Sca-1^+^ BMCs under hypoxic conditions and applied to cultured cardiac fibroblasts isolated from old mice (20 months old). The purity of the fibroblast population was determined with DDR2 staining (Supplementary Figure 2A, 2B). The migration, proliferation, differentiation, and senescence of these cultured cells was then evaluated (Figure 1). Cell migration was evaluated using the scratch wound assay on old cardiac fibroblasts cultured for 48 hours, with CM from young or old Sca-1^+^ BMCs. Fibroblast migration was enhanced when cultured in Y-Sca-1^+^CM, compared to those in O-Sca-1^+^CM or serum-free media (p<0.01) (Figure 1A, 1B). Y-Sca-1^+^CM also increased fibroblast proliferation compared to O-Sca-1^+^CM (p<0.01) (Figure 1C, 1D). In contrast, O-Sca-1^+^ CM decreased cell migration (p<0.01) and proliferation (p<0.01), compared to serum-free media-treated.

To evaluate the differentiation of fibroblasts treated with CM, α-SMA^+^ stress fiber formation was studied. Old cardiac fibroblasts, treated with Y-Sca-1^+^ CM, yielded increased numbers of α-SMA^+^ cells (p<0.01) (Figure 1E, 1F), while O-Sca-1^+^ CM reduced the number of α-SMA^+^ cells compared to Y-Sca-1^+^ CM-treated and serum-free media-treated old cardiac fibroblasts (p<0.01, p=0.015) (Figure 1E, 1F). To further evaluate the effect of CM on fibroblast differentiation to myofibroblasts, a gel contraction assay was performed. Gel containing old cardiac fibroblasts was incubated in Y-Sca-1^+^ CM and showed greater contraction, than O-Sca-1^+^ CM or serum-free media-treated cells (p<0.01) (Figure 1G, 1H).

Proliferation, migration, and differentiation are all indicators of fibroblast function which deteriorate with age, but they are not directly used to evaluate cellular aging. To determine whether aged fibroblasts could be rejuvenated, senescence associated β-galactosidase (SA-β-gal) staining was employed. After old cardiac fibroblasts were cultured with CM for 48 hours, the percentage of SA-β-gal^+^ cells was lower in fibroblasts cultured with Y-Sca-1^+^ CM, compared to O-Sca-1^+^ CM or serum-free media (p<0.01) (Figure 1I, 1J), suggesting the young CM is likely able to rejuvenate aged fibroblast function.

To further confirm that biological molecules present in CM are responsible for restoring cellular function and cellular rejuvenation, CM was heat inactivated. When CM was heat-inactivated, all beneficial phenotypic effects of Y-Sca-1^+^ CM were lost (Supplementary Figure 3A–3H). Thus, bioactive molecules in CM from Y-Sca-1^+^ BMCs play an important role in improving cardiac fibroblast function.

### Autophagy is reduced in old BMCs

**Figure 2:**
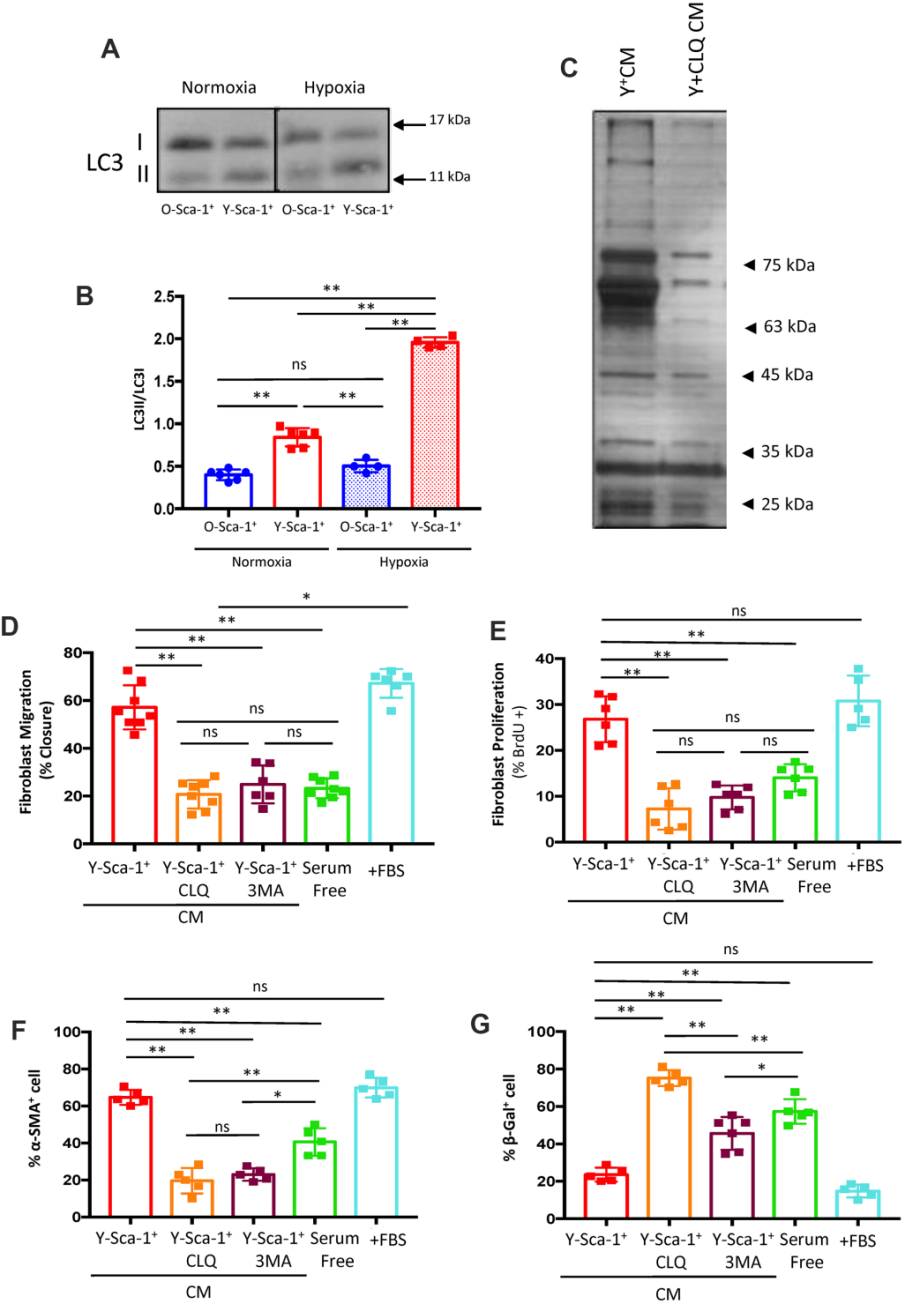
**Autophagy in Y-Sca-1^+^ BMCs is associated with beneficial paracrine effects on old cardiac fibroblasts.** (**A**) LC3II and LC3I were measured in Y-Sca-1^+^ and O-Sca-1^+^ bone marrow cells (BMCs) under normoxic (n=6) and hypoxic (n=4) conditions. (**B**) Quantification of LC3II/I protein intensities shown in panel (**A**). (**C**) Conditioned medium (CM) was harvested from Y-Sca-1^+^ (Y^+^CM) and Y-Sca-1^+^+ chloroquine (CLQ, Y^+^CLQ CM). Proteins were separated by 10% SDS-PAGE. Representative image of silver-stained gel from Y^+^CM±CLQ. (**D**–**G**) CM was collected from Y-Sca-1^+^ BMCs treated with chemical inhibitors of autophagy, CLQ and 3-Methyladenine (3MA), and added to cultured cardiac fibroblasts from old mice (>20 months old) for 48 hours. Treatments are abbreviated as: Y-Sca-1^+^ CLQ and Y-Sca-1^+^ 3MA, serum free (serum-free media), +FBS (complete growth media). (**D**) Percent wound closure (after completing scratch wound assay) was measured using ImageJ. (**E**) Proliferation was assessed as the percentage of BrdU^+^ cells, normalized to total cell number. (**F**) Differentiation was determined as the percentage of α-SMA^+^ cells, normalized to total cell number. (**G**) Senescence was measured as the percentage of β-galactosidase^+^ cells, normalized to total cell number. Data with multiple groups were analyzed using one-way ANOVA, while data with two groups were analyzed by t-test. **p≤0.01; ns: not statistically significant.

Autophagy is activated under stress conditions, such as starvation and hypoxia [8], and this stress response is diminished with age. To investigate the effect aging has on BMC autophagy, BMCs were cultured under normoxic or hypoxic conditions for 24 hours, as described above. When cultured in normoxic conditions, the LC3bII to LC3bI ratio was elevated in Y-Sca-1^+^ compared to O-Sca-1^+^ BMCs (p<0.01), indicating age-related differences in basal autophagy (Figure 2A, 2B). This trend was accentuated when BMCs were challenged with hypoxia (Figure 2A, 2B).

To further evaluate the role of autophagy on the secretome of Y-Sca-1^+^ BMCs, chloroquine (CLQ), a pharmacological inhibitor that prevents binding of the autophagosome to the lysosome, was administered. Gross protein content from Y-Sca-1^+^CM and Y-Sca-1^+^CLQ CM was assessed by silver staining, which showed that inhibition of autophagy (Y-Sca-1^+^CLQ CM) dramatically altered the secretome composition of Y-Sca-1^+^ BMCs (Figure 2C).

### Blocking autophagy in young Sca-1^+^ BMCs reverses the beneficial paracrine effects on aged cardiac fibroblasts

To better understand the role that autophagy has in paracrine signaling, autophagy was blocked in Y-Sca-1^+^ BMCs using chemical inhibitors of autophagy, CLQ and 3-Methyladenine (3MA), prior to collection of CM. Trypan blue staining was performed to confirm that cell viability was maintained after CLQ and 3MA treatment (Supplementary Figure 4). Fibroblasts were cultured in serum-free or full growth (+FBS) medium as negative and positive controls, respectively, and cellular function was assessed via changes in: migration, proliferation, differentiation, and senescence (quantification and representative images are shown in Figure 2D–2G and Supplementary Figure 5A–5D, respectively). CLQ and 3MA treatment effectively reversed any beneficial effect that CM from Y-Sca-1^+^ BMCs had on fibroblast function and rejuvenation, as migration, proliferation and differentiation were decreased, but senescence was increased (p<0.01) (Figure 2D–2G).

**Figure 3:**
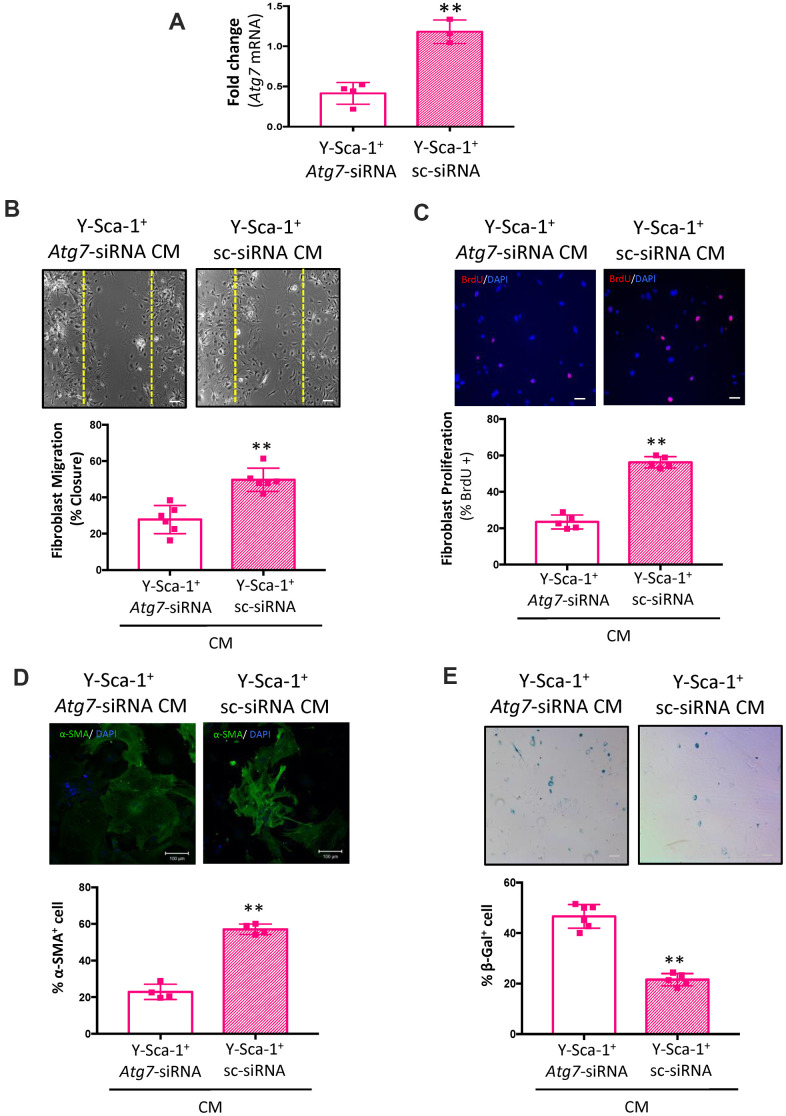
***Atg7* knockdown in Y-Sca-1^+^ BMCs blocks paracrine stimulation of old cardiac fibroblasts.** (**A**) *Atg7* expression was measured in Y-Sca-1^+^ bone marrow cells (BMCs), treated with *Atg7*-siRNA or sc-siRNA, by RT-qPCR. n=3-4. (**B**–**E**) Quantification and representative images from (**B**) scratch wound assay, (**C**) proliferation assay, (**D**) stress fiber formation or (**E**) β-galactosidase staining of old cardiac fibroblasts, treated with Y-Sca-1^+^ *Atg7*-siRNA CM or Y-Sca-1^+^ sc-siRNA CM for 24-48 hours. Dashed yellow line in (**B**) indicates the wound edge at 0 hours. Scale bar represents 100 μm. Data analysis was carried out by t-test. Data presented as mean ± SEM; n=4-6; **p≤0.01.

To further examine the effect of autophagy on secretion of factors from Y-Sca-1^+^ BMCs, autophagy was inhibited using *Atg7*-siRNA or Scrambled-siRNA (sc-siRNA) prior to CM collection. *Atg7* knockdown was confirmed using RT qPCR (Figure 3A). Compared to cells that received scrambled siRNA, the CM produced from Y-Sca-1^+^ BMCs treated with *Atg7*-siRNA reduced old cardiac fibroblast migration, proliferation, and differentiation, but increased cell senescence (p<0.01) (Figure 3B–3E). Therefore, from using two chemical inhibitors of autophagy and gene-specific knockdown of *Atg7*, these data show that autophagy underlies paracrine signaling in Y-Sca-1^+^ BMCs.

### Autophagy regulates extracellular vesicle secretion and/or cargo in Sca-1^+^ BMCs

**Figure 4:**
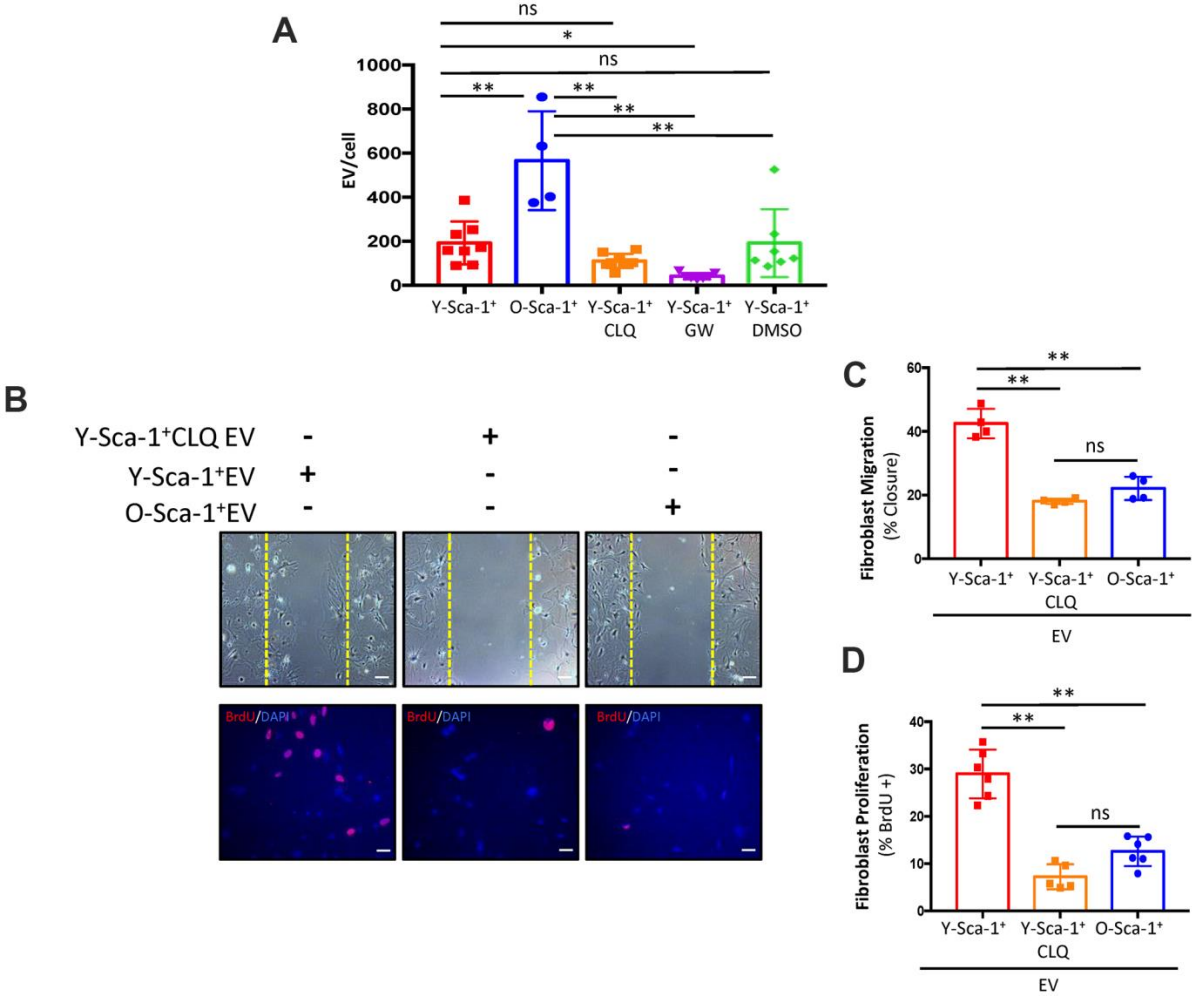
**Autophagy is associated with EV secretion and/or cargo in Sca-1^+^ bone marrow cells.** (**A**) Extracellular vesicle (EV) particle concentration isolated from Y-Sca-1^+^ CM±CLQ, ±GW4869, ±DMSO and O-Sca-1^+^ conditioned medium (CM). EV production was inhibited in Y-Sca-1^+^ bone marrow cells (BMCs) using GW4869 (GW). Autophagy was inhibited using chloroquine (CLQ). (**B**–**D**) EVs were purified from CM of the same treatment groups, and added to cultured old cardiac fibroblasts. Treatment groups are abbreviated as: Y-Sca-1^+^ EV, Y-Sca-1^+^ CLQ EV and O-Sca-1^+^ EV. (**B**) Representative images from scratch wound and proliferation assays with old fibroblasts treated with Y-Sca-1^+^ EV, Y-Sca-1^+^ CLQ EV and O-Sca-1^+^ EV for 48, or 24 hours, respectively. Dashed yellow line indicates the wound edge at 0 hours. (**C**) Percent wound closure (after completing the scratch wound assay) was measured using ImageJ. (**D**) Percentage of BrdU^+^ cells, normalized to total cell number. Scale bars represent 100 μm. Data analysis used one-way ANOVA. Data presented as mean ± SEM; n=4-8. *p≤0.05; **p≤0.01; ns: not statistically significant.

Autophagy participates in secretory pathways that are EV-dependent and/or independent [18, 19]. To evaluate the effect of autophagy on EV quantity, EV concentration and size were assessed using nanoparticle tracking analysis. O-Sca-1^+^ BMCs secreted more particles compared to Y-Sca-1^+^ BMCs (Figure 4A), a phenomenon that has also been observed in senescent cells [20–22]. No significant differences in particle sizes were detected among the groups (Supplementary Figure 6). Treatment of Y-Sca-1^+^ BMCs with GW4869 (a neutral sphingomyelinase inhibitor that blocks exosome generation, referred to here as GW) significantly reduced EV secretion. Interestingly, inhibition of autophagy by CLQ (Y-Sca-1^+^CLQ) did not reduce particle secretion under hypoxic conditions (Figure 4A). To determine whether inhibition of autophagy leads to functional changes in secreted EVs, old cardiac fibroblasts were cultured for 24 hours with EVs isolated from Y-Sca-1^+^ CM, Y-Sca-1^+^CLQ CM and O-Sca-1^+^ CM (referred to as Y-Sca-1^+^ EV, Y-Sca-1^+^CLQ EV and O-Sca-1^+^ EV, respectively). The cell migration assay showed that 48 hours treatment with Y-Sca-1^+^ EVs enhanced old cardiac fibroblast migration, compared to O-Sca-1^+^ EV treatment (p<0.01) (Figure 4B, 4C). This phenotype was not observed in old cardiac fibroblasts treated with Y-Sca-1^+^CLQ EVs. Similarly, when treated with Y-Sca-1^+^ EVs, proliferation of old cardiac fibroblasts was greater than when treated with O-Sca-1^+^ EVs (p<0.01), and inhibition of autophagy in Y-Sca-1^+^ BMCs by CLQ (Y-Sca-1^+^CLQ EV) blocked this effect (Figure 4B, 4D). As autophagy inhibition precluded the beneficial effects of Y-Sca-1^+^ EVs on fibroblast function, these results thus underscore the relationship between autophagy and EV-dependent paracrine signaling.

### Y-Sca-1^+^ BMCs improve old cardiac fibroblast function, in part by TGF-β1

**Figure 5:**
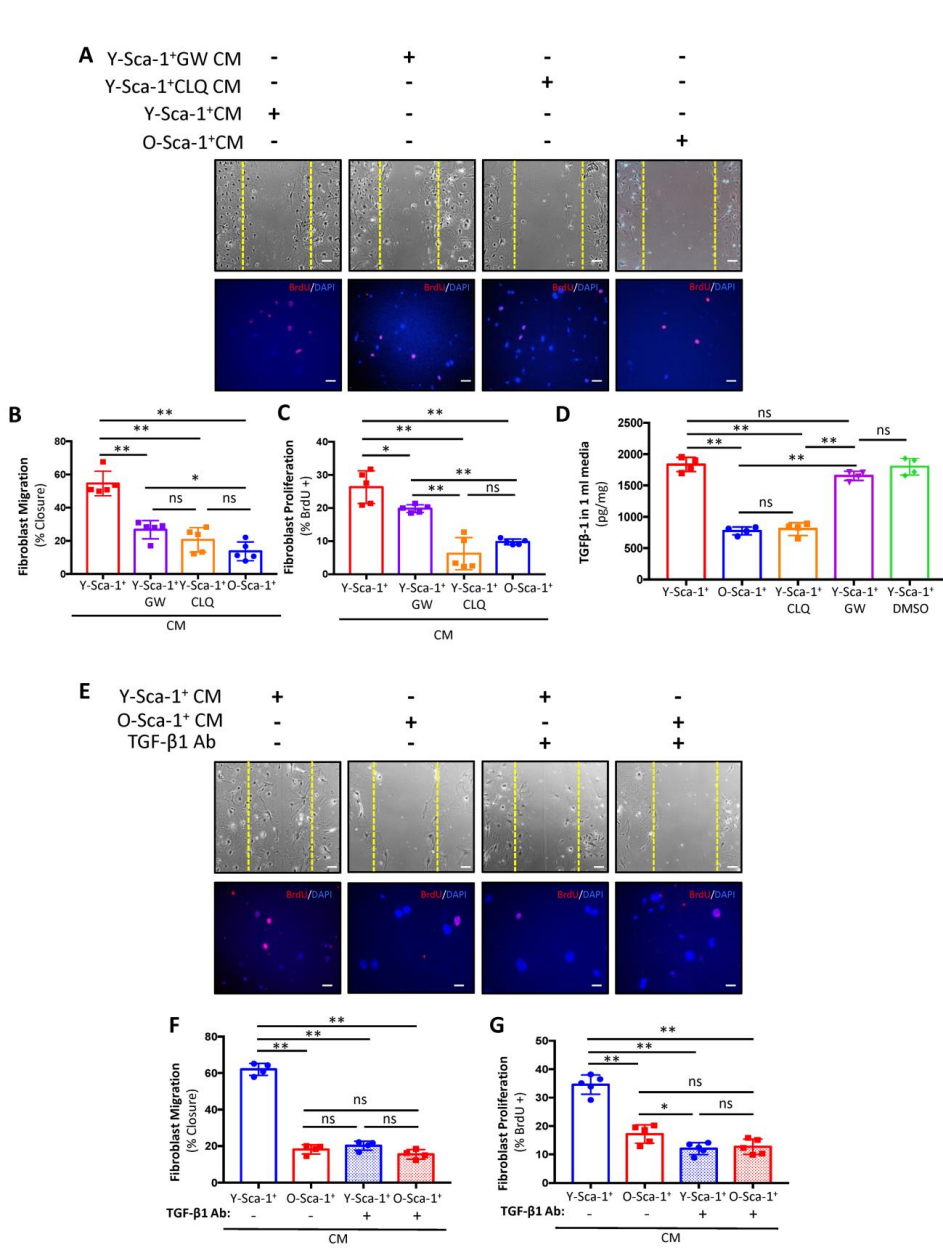
**Y-Sca-1^+^ BMCs enhance old cardiac fibroblast function via autophagy-dependent TGF-β1 secretion.** (**A**) Y-Sca-1^+^ bone marrow cells (BMCs) were treated with pharmacological inhibitors of autophagy (chloroquine, CLQ) and extracellular vesicle (EV) secretion (via GW4869). Conditioned medium (CM) was then collected and added to cultured cardiac fibroblasts isolated from old mice. Treatments are abbreviated as: Y-Sca-1^+^ CM±CLQ, Y-Sca-1^+^ CM±GW4869 or O-Sca-1^+^ CM. Representative images from scratch wound and proliferation assays after old cardiac fibroblasts were treated with CM from Y-Sca-1^+^ CM±CLQ, ±GW4869 and O-Sca-1^+^ CM for 48, or 24 hours, respectively. (**B**) Percent wound closure (after completing the scratch wound assay) was measured using ImageJ. Dashed yellow line indicates the wound edge at 0 hours. (**C**) Percentage of BrdU^+^ cells, normalized to total cell number. (**D**) TGF-β1 levels were measured in CM using ELISA. (**E**) Representative images from scratch wound and proliferation assays after old fibroblasts were treated with CM in the presence of TGF-β1 blocking antibody (Ab) for 48 hours. Treatment groups are abbreviated as: Y-Sca-1^+^ CM± TGF-β1 Ab and O-Sca-1^+^ CM± TGF-β1 Ab. Dashed yellow line indicates the wound edge at 0 hours. (**F**) Percent wound closure (after completing the scratch wound assay) was measured using ImageJ. (**G**) Percentage of BrdU^+^ cells, normalized to total cell number. Scale bars represent 100 μm. One-way ANOVA used to analyze data. n=3-4 *p≤0.05; **p≤0.01; ns: not statistically significant.

Overall, the above data indicate that EV secretion influences cardiac fibroblast function, and their release and/or cargo from bone marrow cells is dependent on autophagic pathways. To further dissect the importance of autophagy on EV-dependent and independent signaling, Y-Sca-1^+^ BMCs were treated with GW4869 to inhibit exosome secretion during generation of the CM (Y-Sca-1^+^ GW CM). When old cardiac fibroblasts were treated with Y-Sca-1^+^GW CM, migration was lower compared to Y-Sca-1^+^ CM treatment (p<0.01) (Figure 5A, 5B). This reduction was equivalent to that observed in the Y-Sca-1^+^CLQ CM treatment group (p=0.3). Proliferation was also reduced after treatment with Y-Sca-1^+^GW CM, compared to the Y-Sca-1^+^ CM-treated group (p=0.02) (Figure 5A, 5C). However, inhibition of autophagy (Y-Sca-1^+^CLQ CM) had a more potent effect on repressing cardiac fibroblast proliferation than inhibition of EV production (p<0.01) (Figure 5A, 5C) suggesting that EV-independent factors are present that also play an important role in stimulating cardiac fibroblast activity.

One of the candidates in our previous screen [23], and confirmed in this study to be affected by aging in BMCs, is TGF-β1 (Supplementary Figure 1A), which has an established role in fibroblast function and scar formation post-MI [24]. To determine if TGF-β1 is carried in EVs or directly secreted into CM, the levels of TGF-β1 were measured in CM and EVs. Age significantly reduced TGF-β1 levels in CM collected from O-Sca-1^+^ CM (p<0.01), as did CLQ (Figure 5D). Notably, GW did not affect TGF-β1 secretion, thereby representing an EV-independent paracrine signaling mechanism.

To investigate whether TGF-β1 participates in the paracrine effects observed in our previous experiments, old cardiac fibroblasts were pre-treated with TGF-β1 neutralizing antibody (TGF-β1 Ab) for 48 hours, followed by treatment with Y-Sca-1^+^ CM and O-Sca-1^+^ CM with TGF-β1 Ab. Fibroblasts cultured in Y-Sca-1^+^ CM with TGF-β1 Ab showed reduced migration (Figure 5E, 5F) and proliferation (Figure 5E, 5G), compared to the non-TGF-β1 Ab treated group. These data suggest that the beneficial, EV-independent paracrine effects of Y-Sca-1^+^ BMCs were contributed in part by TGF-β1.

### Y-Sca-1^+^ BM reconstitution enhances autophagy in the old heart

**Figure 6:**
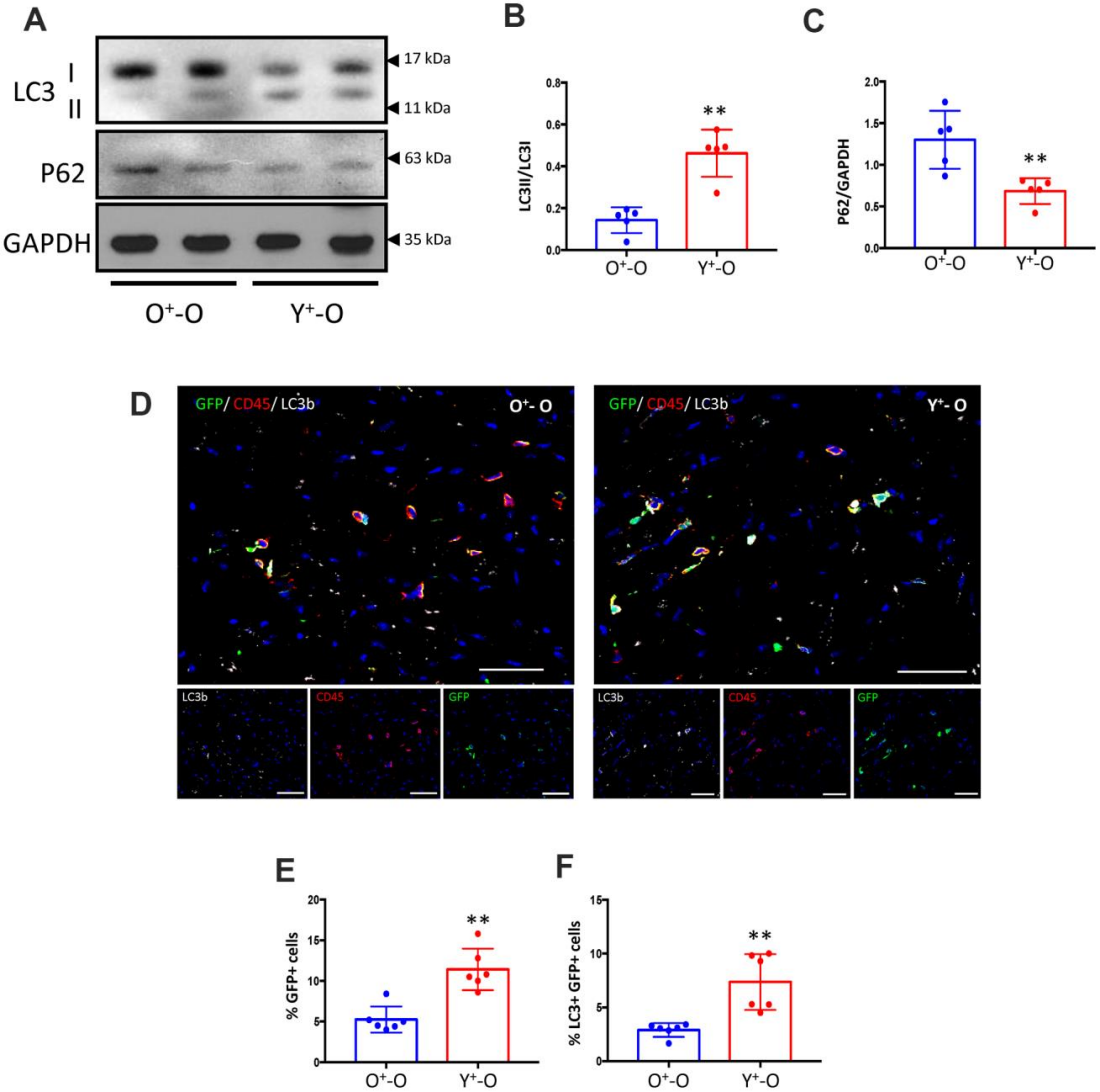
**Heterochronic bone marrow reconstitution by Y-Sca-1^+^ BMCs is associated with enhanced autophagy in the aged mouse heart.** (**A**) LC3I, II and p62 protein levels were measured in old mice hearts, reconstituted with GFP^+^young-Sca-1^+^ (Y^+^-O) or GFP^+^old-Sca-1^+^ (O^+^-O) bone marrow cells (BMCs) by Western blotting. (**B**, **C**) Band intensities shown in panel (**A**) were quantified to measure LC3II/I ratio or p62 levels. Data were normalized to loading control GAPDH. (**D**) Immunofluorescent staining was performed on sections of Y^+^-O and O^+^-O myocardium. LC3b is indicated by yellow, CD45 by red, GFP^+^ BMCs by green, and nuclei by blue. Scale bar represents 50 μm. Percentage of GFP^+^ cells (**E**), or LC3^+^ and GFP^+^ cells (**F**) in panel (**D**) were quantified. Scale bars represent 50 μm. T-test used to analyze data. n=5-6; *p≤0.05; **p≤0.01.

To study the effect of young BM stem cells on autophagy *in vivo*, lethally irradiated old mice were reconstituted using Y- or O-Sca-1^+^ BMCs from GFP^+^ mice. Three months after BM reconstitution, protein analysis of cardiac tissue showed that old BM replacement with young-Sca-1^+^ cells (Y^+^-O) increased the LC3II/LC3I ratio (p<0.01) and reduced p62 (an ubiquitin-binding scaffold protein) levels (p<0.01), compared to old-Sca-1^+^ (O^+^-O) chimeric mice (Figure 6A–6C). Immunofluorescent staining of heart tissue from Y^+^-O and O^+^-O chimeric mice showed greater numbers of recruited GFP^+^ donor cells (p<0.01) (Figure 6D, 6E). In addition, there were significantly more GFP^+^/LC3^+^ (donor cells) (p< 0.01) in the myocardium of the Y^+^-O group compared to the O^+^-O group (Figure 6F).

Together, the *in vitro* data suggest that Y-Sca-1^+^ BMCs enhance old cardiac fibroblast function in a paracrine manner that requires both EV-dependent and -independent pathways. *In vivo*, Y-Sca-1^+^ BMCs home to the heart at a higher rate and this corresponded to changes in autophagy within the old heart. Therefore, BM donor cell autophagy likely has a role in stimulating cardiac repair via paracrine signaling *in vivo*, but the age-related decline in autophagy of donor cells may limit their beneficial effects.

## DISCUSSION

In the current study, we demonstrated that autophagy in young BMCs plays a major role in altering cardiac fibroblast function isolated from old mice. We restored cellular function in proliferation, migration and differentiation of old fibroblasts using Y-Sca-1^+^ CM and showed that this effect was dependent on autophagy in Y-Sca-1^+^ BMCs via both EV-dependent and independent pathways. Our data show that autophagy regulates the secretion of various factors, including TGF-β1, which acts in an EV-independent manner to enhance old cardiac fibroblast function. The *in vivo* study shows that old BM reconstituted with Y-Sca-1^+^ BMCs migrate to the heart at a higher rate and influence cardiac autophagy, as demonstrated by changes in the LC3II/I ratio and the decline in p62 protein levels. Future examination of autophagy and EV-dependent and independent paracrine signaling should be evaluated in the context of cell therapy to determine how the secretome modulates the reparative processes of the local environment in the ischemic, aged heart.

Despite their therapeutic potential, the efficacy of stem cell-based therapies in old individuals remains a major challenge. Dysfunctional autophagy during aging has been reported in various tissues [12], which may affect cellular homeostasis and function. As a result, autophagy has recently received significant attention in the aging field, where it has been shown to be a key cellular process impacting the aging phenotype for multiple tissues. Previous studies have shown that autophagy is highly regulated in the central neural system, where it plays an important role in regulating brain function. Alterations of autophagy can lead to pathological phenotypes in neurons associated with neurodevelopmental and neurodegenerative diseases [25]. Autophagy has also been demonstrated to be an important regulator of lung development and morphogenesis [26]. With respect to cardiac tissue, autophagy is one of the mechanisms involved in tissue repair and regeneration, in which its activation via caloric restriction, or mTOR activity inhibition (through rapamycin administration), improves heart functions in aged animal models [27, 28]. Additionally, Beclin-1 (an initiator of autophagosome formation) is elevated during ischemia/reperfusion, stimulating autophagic flux and subsequent scar size reduction [29]. A study using a zebrafish injury model demonstrated that autophagosomes accumulated at the site of cardiac tissue amputation, which was followed by autophagy activation during early stages of cardiac regeneration [30]. The beneficial effect of BM reconstitution with Y-Sca-1^+^ BMCs on subsequent cardiac function after an MI has also been previously reported [6, 31], yet the mechanisms responsible were unclear. Our findings align with the general observation that autophagy declines with age [12], as O-Sca-1^+^ BMCs were unable to activate autophagy in response to hypoxia. Since paracrine signaling is currently the primary mechanism of cell therapy in heart repair [32], a potential limiting factor of autologous cell therapy in the elderly would be defective autophagy.

The roles of cardiac fibroblasts in heart development and function have been extensively studied [33]. Forty to 60% of the heart’s cellular mass consists of fibroblasts [34], which serve as structural support for the organ via their interaction with cardiomyocytes. However, upon myocardial injury, the ensuing mechanical and inflammatory stimulation on the fibroblasts trigger their differentiation into myofibroblasts, which are not part of the regular heart tissue. These myofibroblasts are highly sensitive to chemokine release, produce stress fibers, and migrate to the injury site [35]. A key inflammatory cytokine, responsible for fibroblast differentiation into myofibroblasts, as well as subsequent proliferation, is TGF-β [36]. This process is then followed by increasing smooth muscle actin expression and eventual collagen deposition [37]. We demonstrated that isolated cardiac fibroblasts proliferated in young Sca-1^+^ CM and displayed increased α-SMA expression. Fibroblast cell function was evaluated by gel contraction assay, which showed that young Sca-1^+^ CM-treated fibroblasts had greater gel contractility, suggesting those fibroblasts expressed a myofibroblast phenotype. Additionally, our findings suggested that TGF-β secreted into the CM by young Sca-1^+^ cells is likely responsible for this fibroblast-myofibroblast differentiation. These findings are therefore in agreement with other studies reporting TGF-β stimulation of fibroblasts to differentiate into myofibroblasts, with associated increased contractility [36], such as Anitua et al., who reported that growth factor-rich plasma (containing platelet-derived growth factor, fibroblast growth factor, insulin-like growth factor, etc.) increases fibroblast proliferation and production of VEGF, TGFβ and collagen type I [38].

It has been reported that the high α-SMA levels in cardiac fibroblasts may not be the sole cause for the contractile ability of myofibroblasts [39]. Gel contraction assay confirms that young Sca-1^+^ CM increases fibroblast contractile abilities, though further investigation is necessary to determine whether that increase is due to high α-SMA levels. Even though α-SMA^+^ cardiac fibroblasts are usually considered a type of active myofibroblast, with pro-fibrotic tendencies, including collagen production, that are often considered detrimental, owing to the inability of cardiomyocyte self-replenishment, they may also have beneficial effects for heart repair and healing. These benefits stem from their contractile abilities, as well as being viable, metabolically active cells maintaining the infarct area as a living tissue [40]. Myofibroblasts are also vital for extra cellular matrix (ECM) remodeling and scar formation post-MI, and low numbers in the scar area leads to poor ECM remodeling, along with inadequate healing. The contractile ability of myofibroblasts prevents infarct scar progression, heart dilation and ventricular wall thinning, which elevates post-MI heart failure prevalence [41]. Aging is one of the factors reducing fibroblast proliferation and differentiation to myofibroblasts. Owing to the aforementioned vital roles they play in post-MI wound healing, dampened proliferation would thus have severe consequences [42]. Here, we showed that Y-Sca-1^+^ CM improved old fibroblast function, and these beneficial paracrine effects were autophagy dependent. Restoration of cardiac fibroblast function may contribute to the prevention of heart failure.

Previous studies have shown that autophagy-dependent secreted factors stimulated fibroblast proliferation and migration through autocrine and paracrine signaling [10]. The cellular secretome can be directly excreted, or packaged into EVs that are then secreted into the extracellular space [19, 43]. Extensive research has been done to investigate the role of autophagy in regulating EV secretion [11, 44]. When placed under stress, such as during aging or after MI, cells may bypass conventional autophagic pathways and release the secretome via EVs [45]. Cellular senescence has been suggested to increase EV secretion [46], which agrees with our observation that O-Sca-1^+^ BMCs secrete more EVs compared to Y-Sca-1^+^ BMCs.

The relationship between autophagy and BMC aging, in particular with respect to associated underlying pathways, is still ambiguously defined. It is worth noting, though, that a number of studies have shown that impairments in mitochondrial function, as well as decreased mitophagy (autophagy-mediated mitochondrial degradation) serve as major mechanisms controlling BMC aging. For instance, Zhang et al. showed that impairment of mitochondrial NADH dehydrogenase (ubiquinone) iron-sulfur protein 6 is a putative accelerator of adult BMC aging, and is associated with excess ROS accumulation and upregulation of cell senescence [47]. Other studies have also examined the role mitochondria efficiency plays in delineating hematopoietic stem cells (HSCs) pluripotency potential and differentiation abilities, where mitophagy plays an important role in maintaining HSC quiescence and proper hematopoietic differentiation. It is thus no surprise, then, that disruption of autophagy components results in early lethality from severe anemia [48]. This current study has provided some support to those findings by demonstrating that deterioration of autophagy with aging negatively impacts the paracrine effects of BMCs. However, the causative relation between BMCs and autophagy needs further investigation. With respect to the change in components within CM extracted from young and old BMCs, multiple paracrine factors are likely to contribute to that comprehensive effect observed from young Sca-1^+^CM on old cardiac fibroblasts. The upregulation of angiogenic cytokines, such as Erb-B2 receptor tyrosine kinase 4 (ERBB4) [49], and the down-regulation of pro-senescence cytokines which affect senescence-associated secretory phenotype (SASP) [50], are one of several possibilities. Indeed, ERBB4 rejuvenated aged mesenchymal stem cells (MSCs) and protected them from oxidative stress-induced senescence, while mitochondria-derived peptides increased mitochondrial respiration and certain SASP factors. However, secretome proteomics or mass spectrometry would be required to reveal the underlying secreted factors responsible for our observations. Furthermore, the efficiency of the secretory pathway itself is another factor possibly playing a role in the differences between young and old Sca-1^+^CM. Future investigations should also aim to reveal the underlying pathways within BMC CM responsible for the paracrine effects. One possibility is the Rap1 (also known as telomeric repeat-binding factor 2-interacting protein 1)/NF-κB pathway. Indeed, studies have shown that cytokine and growth factor secretion is mediated by that pathway in MSCs, where it is active in pro-inflammatory settings, such as the aged microenvironment. Absence of Rap1 in BM-MSCs led to downregulation of NF-κB activity, accompanied by reduced pro-inflammatory paracrine cytokines [51], On the other hand, autophagy activation suppresses NF-κB signaling and controls the inflammatory response during injury [52]. Nevertheless, details regarding the age-related role of autophagy with respect to the types and abundances of paracrine factors acting on effector cells in the heart during repair should be fleshed out in future studies.

In conclusion, our data indicate that autophagy regulates secretion patterns of BMCs via EV-dependent and -independent pathways, which improves old fibroblast cell function. These beneficial paracrine mechanisms are lost as BMCs age. Restoring autophagy in old BMCs could improve healing of the heart after MI. Using an *in vivo* model of BM reconstitution, we showed that recruitment of young donor BMCs to the old heart led to increased autophagy in the heart. Our *in vitro* findings suggest that paracrine factors such as TGF-β1 could be responsible for activation of cardiac fibroblasts to enhance cardiac repair and function restoration as we previously demonstrated [53]. These findings provide the foundation for further translational studies to identify treatments to improve autophagy in old stem cells and advance stem cell therapy development.

## MATERIALS AND METHODS

### Cardiac fibroblast isolation and culture

Old cardiac fibroblasts were isolated from mice, age 18-22 months, as previously described [54]. Briefly, the heart was dissected, and the atria removed. The minced heart was digested in 0.2% collagenase II (#LS004174-Worthington) for 10 min in a shaking 37° C incubator. After digestion, the cell suspension was transferred into a tube containing Iscove's Modified Dulbecco's Medium (IMDM) + 10% FBS. The digestion and transfer of the cell suspension was repeated 3 times. The cell suspension tube was then centrifuged at 1600 rpm for 5 min at room temperature., and the resulting pellet of cells were resuspended in IMDM+10% FBS, then passed through a sterile 40-μm cell strainer into a 10 mm culture dish. The media was changed after 3 hours to remove non-adherent cells. Fibroblasts were used for experiments on passage two.

### Animals and bone marrow reconstitution

The Animal Care Committee of the University Health Network approved all experimental procedures, which were carried out according to the Guide for the Care and Use of Laboratory Animals (National Institutes of Health, Bethesda, MD, USA). Young (2-3 month) or old (18-22 month) donor BM was flushed from tibiae and femurs of C57BL/6-Tg-green fluorescent protein (GFP) mice. Sca-1^+^ cells were isolated, as described previously [6], using an immunomagnetic activated cell sorting kit (Stemcell Technologies). Old (18-22 months) female C57BL/6 mice were lethally irradiated (9.5 Gy) and reconstituted with freshly isolated old or young Sca-1^+^ cells through tail vein injection. Three months later, reconstituted mice were euthanized with general anesthetic (ventilated on 5% isoflurane for 5 minutes) followed by the secondary method, which is exsanguination under anesthesia. Dissected heart tissue was prepared for histology and molecular analysis.

### CM production and cellular treatments

Y- or O-Sca-1^+^ cells were isolated, as described above, and cultured under hypoxia (1% O_2_) and serum-free medium for 24 hours. CM was collected and centrifuged at 3000 g for 15 minutes to remove any cellular debris. Supernatant were collected and stored at -80° C for future use. CM and serum-free IMDM were used to treat old fibroblasts in 2:1 ratio, respectively. CM heat inactivation was achieved by placing it in boiling water bath for 5 minutes. To block autophagy, Chloroquine (CLQ) (#C6628, Sigma Aldrich) or 3-Methyladenine (3MA) (#M9281, Sigma Aldrich) were used at 50 μg/ml concentration for 2 hours, and 10 mM concentration for 3-4 hours, respectively. EV secretion was inhibited using GW4869 (#13127, Cayman Chemical) at 20 μM, for 24 hours. TGF-β1 Ab (1 μg/mL) (#MAB240, R&D Systems) was used to neutralize TGF-β1 protein in CM.

### Small interfering RNA (siRNA) transfection

The nonspecific siRNA oligonucleotides (scrambled siRNA) and siRNA oligonucleotides targeting mouse *Atg7* were purchased from OriGene (#SR427399). siRNA was transfected into Y-Sca-1^+^ BMCs using Lipofectamine 2000 (#11668-027, Invitrogen) according to the manufacturer’s protocol.

### Immunofluorescence staining

Immunofluorescence was conducted, as previously described [54]. Briefly, whole heart tissue was sectioned and fixed with 2% paraformaldehyde (PFA). After blocking, sections were incubated overnight at 4° C with primary antibody, DDR2 (#7555, Santa Cruz), LC3b (#2775, Cell Signaling), GFP (#Ab6673, Abcam), CD45 (#550539, BD Biosciences), and α-SMA (#5228, Sigma). Sections were incubated with fluorescent-conjugated secondary antibody (Invitrogen), followed by application of DAPI (Sigma) to stain nuclei. The sections were viewed and photographed using a Zeiss LSM700 confocal microscope, and the digital images were processed with Zeiss ZEN.

### Western blotting

Western blotting was performed, as previously described [34]. Briefly, 50 μg of lysate protein was heated and applied to SDS-PAGE gels, transferred to nitrocellulose membranes, and then immunoblotted. Antibodies used in this study include LC3b (#2775, Cell Signaling) and Rabbit HRP-conjugated secondary antibodies (#7076, Cell Signaling). Visualization was performed with enhanced chemiluminescence (GE Healthcare). The relative band intensities were quantified using ImageJ.

### Cell migration and proliferation assay

Primary old cardiac fibroblasts were seeded on 35 mm culture dishes. After cells were grown to confluence, the monolayer was scratched in a straight line using a 1 ml pipet tip. Cells were washed to remove debris and dead cells. Serum-free media, CM, or EVs were added to the cells and images were taken at 0, 24 and 48 hours after the scratch. Closure distance was measured using ImageJ, and the percent closure distance was calculated. For the proliferation assay, primary old cardiac fibroblasts were seeded on 24 well culture dishes. The next day, CM, serum-free media, or EVs, were added to the wells for 24 hours. Ten μM of 5-bromo-2'-deoxyuridine (BrdU) was added to the wells for 6-8 hours. At the end of staining, cells were fixed with 2% PFA. BrdU^+^ cells were detected using immunostaining of the BrdU antibody (#ab6326, Abcam). Images were captured using Nikon microscope. Percentages of BrdU^+^ cells were calculated based on the number of DAPI^+^ nuclei.

### Collagen gel contraction assay

Collagen gels were prepared using rat-tail collagen (#354236, BD Biosciences). Old cardiac fibroblasts (4×10^5^) were mixed with the neutralized collagen solution. Aliquots of mixture were placed in a 35 mm culture dish, and allowed to gel at room temperature. The gel was then discharged from the culture plate, and incubated with different CM for 48 hours. Gel size was measured using AlphaImager 2200 software, and presented as gel area.

### Senescence β-galactosidase staining

Primary old cardiac fibroblasts were seeded on 35 mm culture dishes. After cells were grown to 70% confluence, serum-free media or CM was added for 48 hours, followed by β-gal staining using Senescence β-galactosidase kit (#9860, Cell Signaling) per the manufacturer’s instructions. Images were captured using Nikon microscope. The percentages of SA-β-gal^+^ cells were calculated.

### Extracellular vesicle (EV) isolation and quantification

EVs were isolated from CM using ultrafiltration followed by precipitation. CM was centrifuged at 1500 g for 15 min to remove cells, followed by 15 minutes centrifugation at 3,000 g to remove cellular debris. CM was filtered through a 0.22 μm syringe filter, after which it was subjected to ultrafiltration using Amicon 10 kDa MWCO centrifugal filters (#C7719, EMD Millipore). Subsequently, EVs were precipitated overnight using total exosome isolation reagent (Invitrogen), according to the manufacturer’s instructions. EVs were re-suspended in sterile PBS (#D8537, Sigma), then EV size distribution and concentration was measured by nanoparticle tracking analysis using Nanosight NS300 and Nanosight NTA software v.3.2 (Malvern Instruments). Three videos were recorded from each sample for analysis. Total vesicle count per sample was normalized to the number of cells used to generate the CM, yielding an EV/cell value for each sample.

### Enzyme-linked immunosorbent assay (ELISA)

CM was concentrated using Amicon 10 kDa MWCO centrifugal filters (#C7719, EMD Millipore). Fifty μg of protein from concentrated CM or EVs were used to measure the levels of TGF-β1 using ELISA (#MB100B, R&D Systems), following the manufacturer’s instruction.

### RT-qPCR

Total RNA was isolated using TRI-Reagent (Sigma), and cDNA was prepared using NxGen M-MulV Reverse Transcriptase (#30222-1, Lucigen). cDNA expression was analyzed using SensiFAST (#98005, Bioline) SybrGreen. Gene expression was quantified using the ΔΔCt method. All genes are normalized to *Gapdh*. Primers are listed in Supplementary Table 1.

### Statistical analysis

Statistical analyses were performed using unpaired, two-tailed Student’s t test and one-way analysis of variance (ANOVA) following Dunnett *post-hoc* analysis conducted using GraphPad Prism 7.0 software. Data are expressed as mean ±SEM.

## Supplementary Material

Supplementary Figures

Supplementary Table 1
