# Supplementary material for: Age-related defects in autophagy alter the secretion of paracrine factors from bone marrow mononuclear cells
Source: Aging (Albany NY). 2021 Jun 4;13(11):14687–708. doi: 10.18632/aging.203127 (PMC8221303; doi:10.18632/aging.203127)
Supplement: Supplementary Figures [file aging-13-203127-s001.pdf]

SUPPLEMENTARY FIGURES

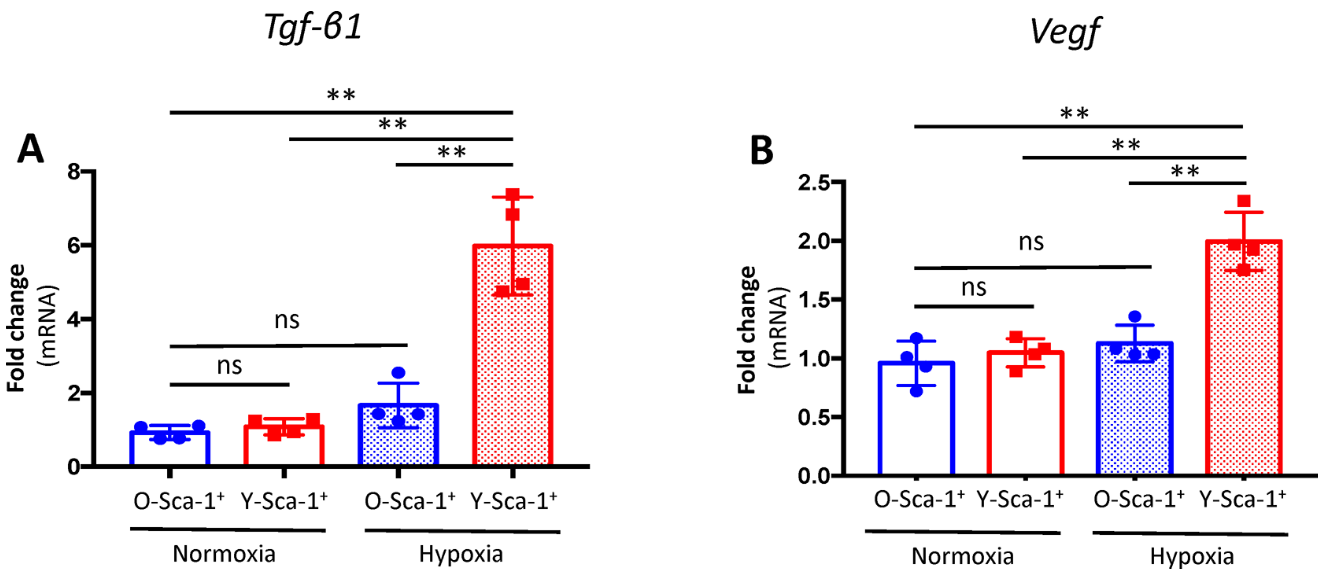

Supplementary Figure 1. *Tgf-β1* (**A**) *Vegf* (**B**) expression were measured in Y-Sca-1⁺ and O-Sca-1⁺ BMCs under normoxic and hypoxic conditions by qRT-PCR (n=4).

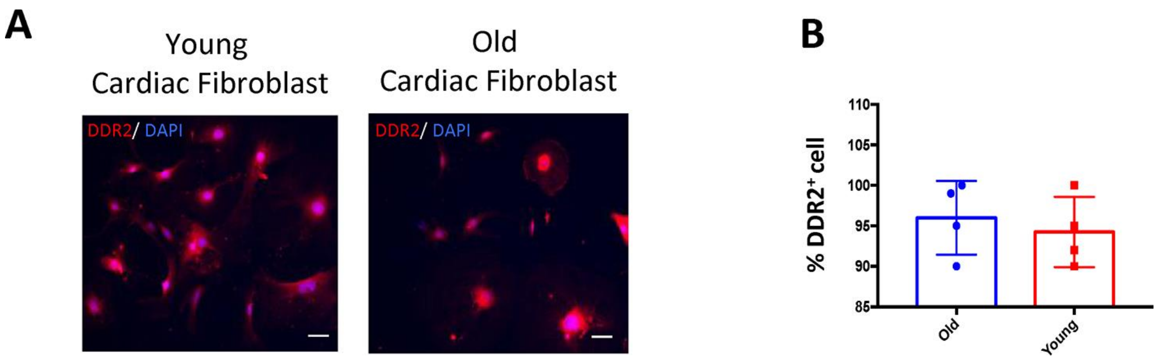

Supplementary Figure 2. Fibroblast purity. (**A**) Immunofluorescent staining was performed on old and young cardiac fibroblasts. DDR2 is shown in red and nuclei are in blue. Scale bar represents 50 μm. (**B**) The percentage of DDR2 positive cells normalized to the total cell number calculated. A t-test was used to analyze data. Data are presented as mean±SE; n=3-6.

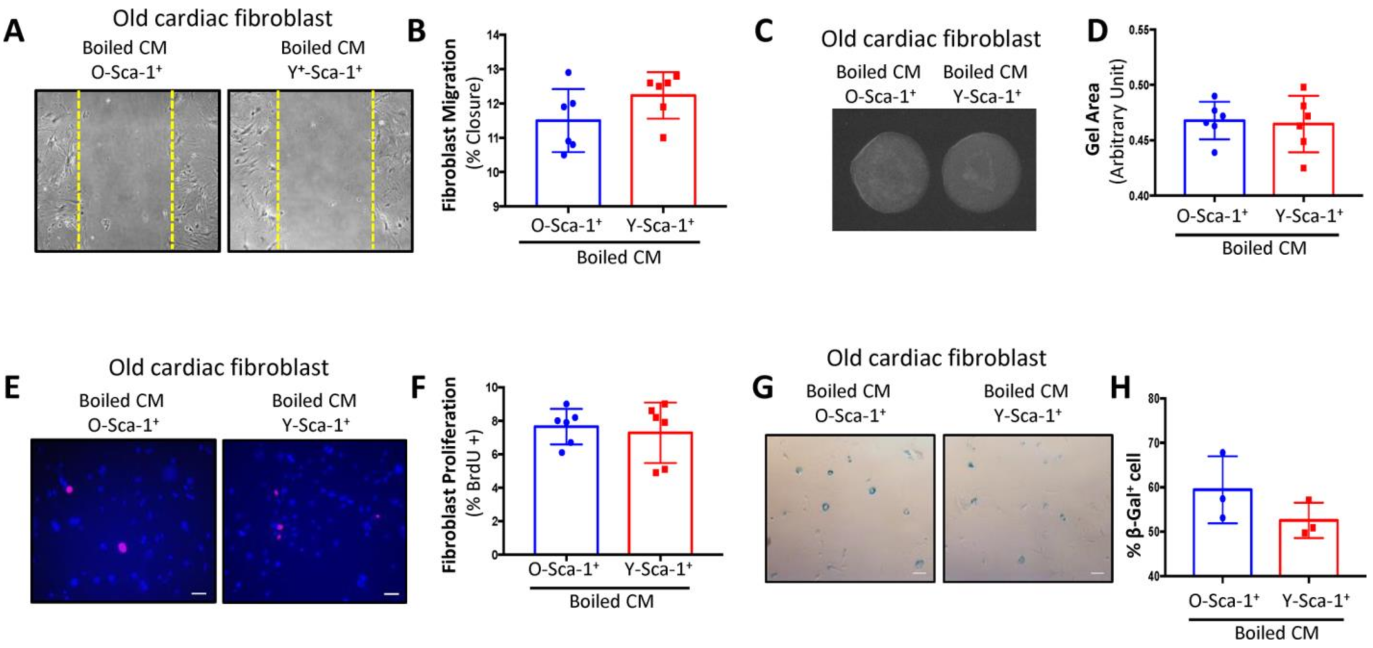

Supplementary Figure 3. Heat inactivated conditioned medium (CM) from Y-Sca-1+ BM cells did not stimulate autophagy in old cardiac fibroblasts. (**A**) Representative images from a scratch wound assay of old cardiac fibroblasts treated with boiled Y-Sca-1+ CM or boiled O-Sca-1+ CM for 48 hours. The dashed yellow line indicates the wound edge at 0 hour. (**B**) The closing distances were measured using ImageJ. (**C**) Representative images of gels from a gel contraction assay of old cardiac fibroblasts treated with boiled Y-Sca-1+ CM or boiled O-Sca-1+ CM for 48 hours. (**D**) The gel area measured using ImageJ. (**E**) Representative images from a proliferation assay of old cardiac fibroblasts related with boiled Y-Sca-1+ CM or boiled O-Sca-1+ CM for 24 hours. (**F**) The percentage of BrdU positive cells to total cell number calculated. (**G**) Representative images of β-galactosidase+ old cardiac fibroblasts treated with boiled Y-Sca-1+ CM or boiled O-Sca-1+ CM for 48 hours. (**H**) The percentage of β-galactosidase+ cells to total cell number calculated. A t-test was used to analyze data. Data are presented as mean±SEM; n=3-6.

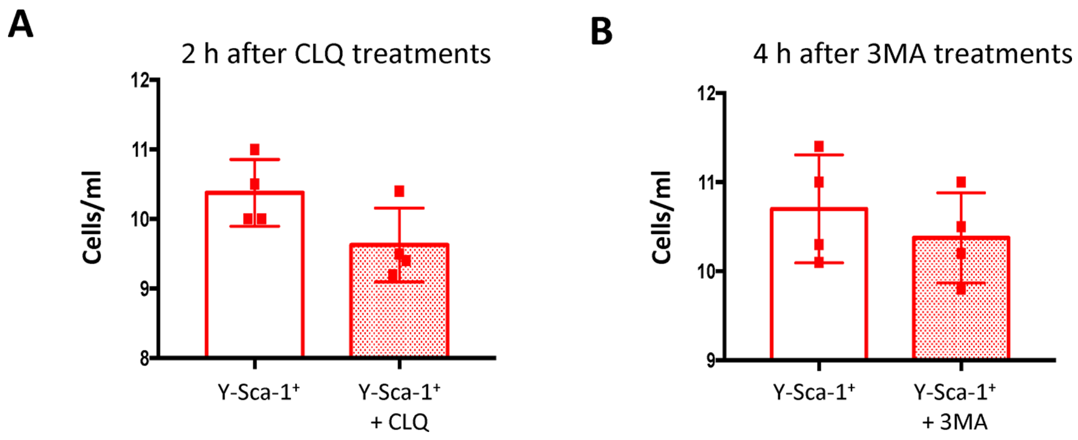

Supplementary Figure 4. CLQ treatment does not induce cell death. (**A**) Y-Sca-1+ BM cells were counted after 2 hours of CLQ treatment and (**B**) Y-Sca-1+ BM cells were counted after 4 hours of 3MA treatment. A t-test was used to analyze the data. Data are presented as mean±SEM.

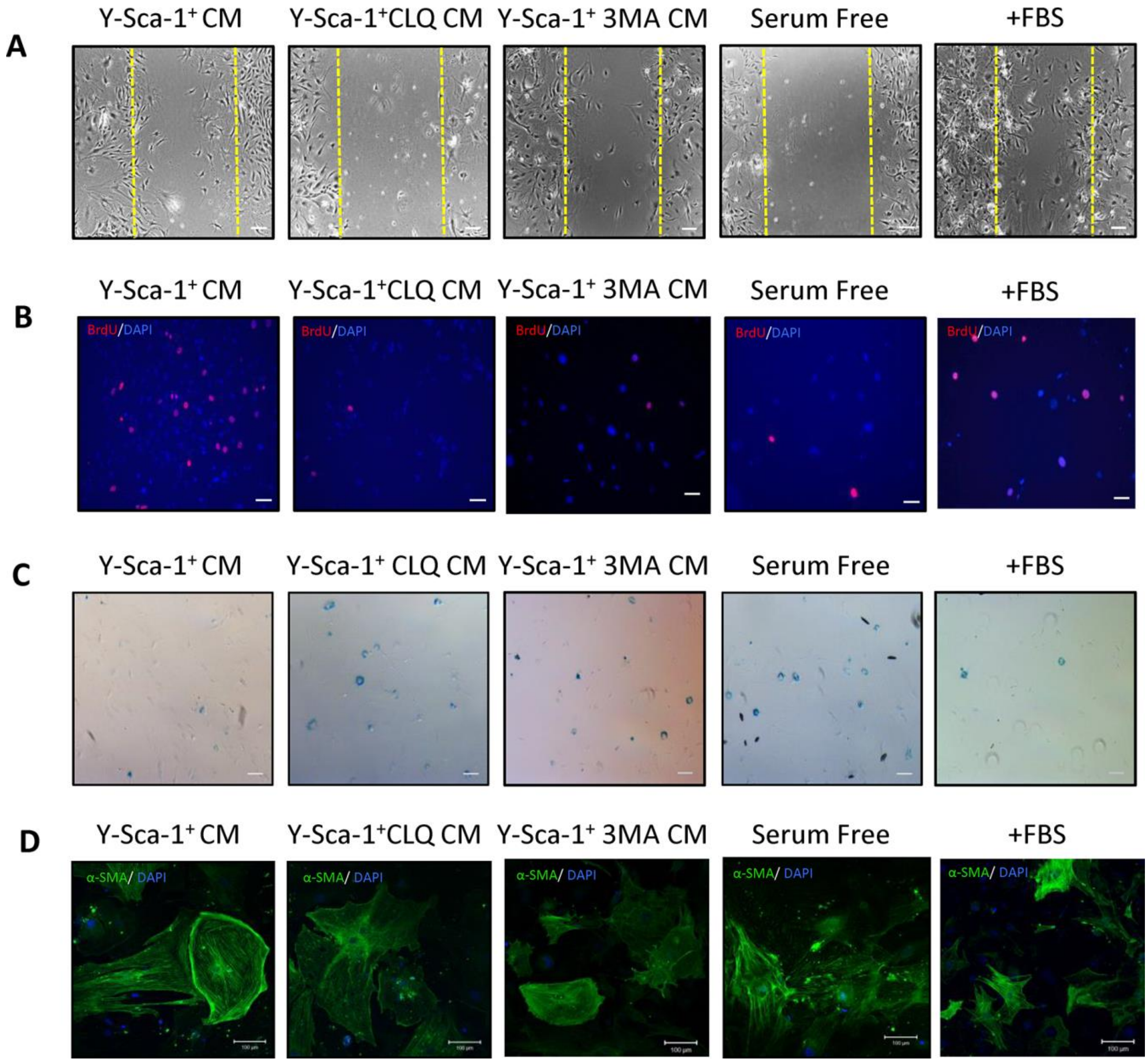

Supplementary Figure 5. Y-Sca-1⁺ BM cell autophagy regulates the beneficial effects of Y-Sca-1⁺ CM on cardiac fibroblast function. (**A**) Representative images from a scratch wound assay of old cardiac fibroblasts treated with Y-Sca-1⁺ CM, Y-Sca-1⁺ CLQ CM, Y-Sca-1⁺ 3MA CM, serum free media or full medium (+FBS) for 48 hours. The dashed yellow line indicates the wound edge at 0 hour. (**B**) Representative images from a proliferation assay of old cardiac fibroblasts treated with Y-Sca-1⁺ CM, Y-Sca-1⁺ CLQ CM Y-Sca-1⁺ 3MA CM, serum free media or full medium (+FBS) for 24 hours. (**C**) Representative images of β-galactosidase⁺ old cardiac fibroblasts treated with Y-Sca-1⁺ CM, Y-Sca-1⁺ CLQ CM Y-Sca-1⁺ 3MA CM, serum free media or full medium (+FBS) for 48 hours. (**D**) Representative images from α-SMA staining of old cardiac fibroblasts treated with Y-Sca-1⁺ CM, Y-Sca-1⁺ CLQ CM Y-Sca-1⁺ 3MA CM, serum free media or full medium (+FBS) for 24 hours. Scale bar represents 100 μm.

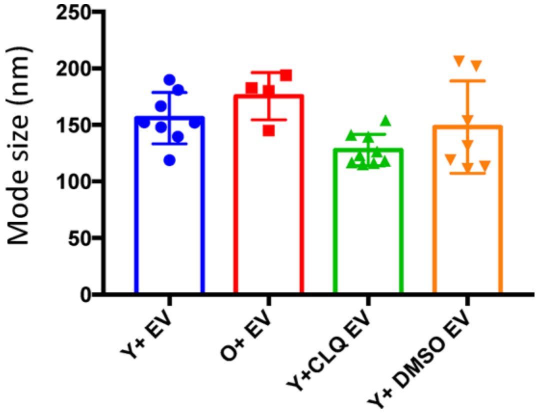

Supplementary Figure 6. EV analysis from Y- and O-Sca-1⁺ BMCs. EV mode size of EVs isolated from CM harvested from Y-Sca-1⁺ (Y⁺ EV); O-Sca-1⁺ (O⁺ EV); Y-Sca-1⁺ +CLQ (Y+CLQ) and, Y-Sca-1⁺ +DMSO (Y+DMSO). One-way ANOVA was used to analyze data. Data presented as means ±SEM; n=4-8.
