# Supplementary material for: Age-related defects in autophagy alter the secretion of paracrine factors from bone marrow mononuclear cells
Source: Aging (Albany NY). 2021 Jun 4;13(11):14687–708. doi: 10.18632/aging.203127 (PMC8221303; doi:10.18632/aging.203127)
Supplement: Supplementary Table 1 [file aging-13-203127-s002.pdf]

SUPPLEMENTARY TABLE

Supplementary Table 1. Oligonucleotide primer sequences.

Gene name	Forward primer	Reverse primer
Tgfβ1	CCTGAGTGGCTGTCTTTTGA	CGTGGAGTTTGTTATCTTTGCTG
Vegf	GGCAGCTTGAGTTAAACGAAC	TGGTGACATGGTTAATCGGTC
Gapdh	CTTTGTCAAGCTCATTTCCTGG	TCTTGCTCAGTGTCCTTGC
Atg7	TTTCTGTCACGGTTCGATAATG	TGAATCCTTCTCGCTCGTACT
